# A Micro-Topography Measurement and Compensation Method for the Key Component Surface Based on White-Light Interferometry

**DOI:** 10.3390/s23198307

**Published:** 2023-10-08

**Authors:** Junying Chen, Boxuan Wang, Xiuyu Chen, Qingshan Jiang, Wei Feng, Zhilong Xu, Zhenye Zhao

**Affiliations:** College of Marine Equipment and Mechanical Engineering, Jimei University, Xiamen 361000, China; chenjunying@jmu.edu.cn (J.C.); 13602210677@163.com (B.W.); jdcxy@126.com (X.C.); fengwei3659@163.com (W.F.); zhilong.xu@163.com (Z.X.); jmuzzy@163.com (Z.Z.)

**Keywords:** key components, surface micro-topography, white-light interferometry, stress concentration

## Abstract

The grinding grooves of material removal machining and the residues of a machining tool on the key component surface cause surface stress concentration. Thus, it is critical to carry out precise measurements on the key component surface to evaluate the stress concentration. Based on white-light interferometry (WLI), we studied the measurement distortion caused by the reflected light from the steep side of the grinding groove being unable to return to the optical system for imaging. A threshold value was set to eliminate the distorted measurement points, and the cubic spline algorithm was used to interpolate the eliminated points for compensation. The compensation result agrees well with the atomic force microscope (AFM) measurement result. However, for residues on the surface, a practical method was established to obtain a microscopic 3D micro-topography point cloud and a super-depth-of-field fusion image simultaneously. Afterward, the semantic segmentation network U-net was adopted to identify the residues in the super-depth-of-field fusion image and achieved a recognition accuracy of 91.06% for residual identification. Residual feature information, including height, position, and size, was obtained by integrating the information from point clouds and super-depth-of-field fusion images. This work can provide foundational data to study surface stress concentration.

## 1. Introduction

High-end equipment is an important indication of a country’s manufacturing level, and key components are the core of high-end equipment [1,2]. Key components work in dynamic situations and often fail owing to fatigue fracture without warning, resulting in catastrophic consequences [3]. Therefore, the key components determine the main functions, life span, and reliability of high-end equipment.

Fatigue failure begins with fatigue crack initiation, extends through crack propagation, and finally fractures abruptly. Fatigue cracks usually arise from the free surface; that is, the fatigue life of a key component heavily depends on its surface condition [4,5,6]. Turning, milling, shaping, grinding, and other machining processes performed during manufacturing leave cutting tracks on the surface, severely damaging the surface integrity. The machining tool cutting tracks have a small-valley radius of curvature, which greatly amplifies the stress level and leads to stress concentration. In addition, other defects, such as machining tool chips being pressed into the machining surface, can also cause stress concentration. Under cyclic-load service conditions, stress concentration will destroy the internal microstructure of the material, resulting in the propagation of cracks. Furthermore, fatigue fractures will occur when the fatigue cracks expand enough that the remaining cross section cannot withstand the applied load [7]. Therefore, key component surface micro-topography is the main influencing factor of fatigue behavior, and its accurate detection and evaluation are more important than those of ordinary part surfaces [5,6].

Numerous scholars [8,9,10,11,12,13,14,15] have adopted various optical microscopic 3D micro-topography measuring instruments to measure the surface of key components and research the influence of surface micromorphology on fatigue properties. These studies used commercial instruments, most of which used ISO25178 parameters to evaluate the surface. However, as mentioned above, the key components are sensitive to stress concentration caused by grinding grooves and surface defects. Still, standardized common instruments do not distinguish whether the surface measured is the surface of the key components or not, and they generally adopt standardized measurement software and hardware. Although the optical method is mainstream for surface micro-topography measurement owing to its noncontact properties and precision, it is also likely to lose signal because the reflected light cannot enter the optical system for imaging if the measured surfaces have a steep angle. However, the side face of the grinding groove is typically steep, which inevitably brings difficulties to optical micro-measurements [16]. In addition, commercial instruments use standard algorithms (generally not open) for data preprocessing, such as filtering, during which key information about the grinding groove may be lost. This loss may result in distortion of the curvature radius, depth, and distribution position of the grinding groove, which are important in the study of surface stress concentration. In addition to the grinding groove, the machining process may also leave defects on the surface, such as residues or burns. Standardized commercial instruments generally cannot distinguish between the defect area and the normal area of the micro-topography measurement point cloud, which brings limitations to the surface stress concentration evaluation of key components. Therefore, the key components need to be customized for the measurement mode. As such, we developed a customized measurement system for key components based on white-light interferometry (WLI).

WLI is widely used to measure surface microscopic 3D topography owing to its non-contact properties, precision, and high efficiency compared to stylus contact measurement or confocal measurement. Thus, it is the most fitting choice for key component surface measurement [17]. Research [9,13,14,15] has been performed to measure the key component surface based on WLI. However, no attention has been paid to the grinding grooves and residues that cause stress concentration. Thus, we researched the measuring difficulty of the grinding groove and the identification of residues to provide more suitable data for the evaluation of key component stress concentrations.

## 2. Measurement Principle of Surface Microscopic Topography Based on WLI

**Figure 1:**
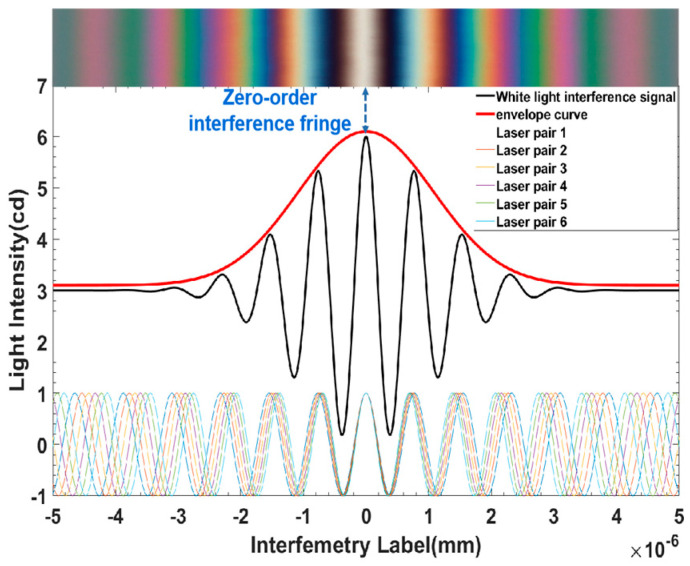
WLI fringe and WLI light-intensity signal.

**Figure 2:**
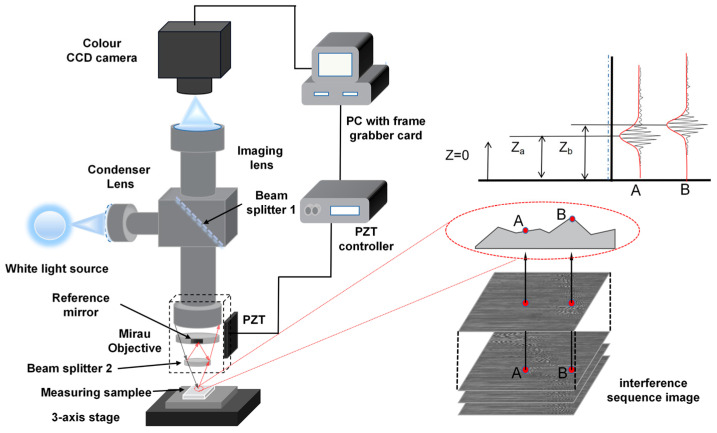
Diagram of WLI principles.

The WLI measurement uses white light as the light source. Owing to the large spectrum width of white light and the short coherence length, the interference phenomenon will occur only when the optical path difference between two light beams is extremely small. This is the physical basis for WLI measurements with high accuracy. When interference occurs, the light of different wavelengths overlaps, and the light intensity reaches its maximum value; the interference fringe at this position is called the zero-level interference fringe, and the interference light intensity takes the shape of an envelope curve, as shown in Figure 1. During the measurement, as shown in Figure 2, the parallel light from the illumination system is divided into two beams after passing through the beam-splitting prism. The beam-splitter plate reflects one beam focus on the measured sample, and the other one casts to the reference plane. The reflected light of these two beams will interfere if they meet the light interference conditions, and interference fringes will be generated. The interference objective lens is mounted on the piezoelectric ceramic transducer (PZT), which can be closed-loop controlled precisely for the vertical displacement drive. The PZT is controlled to move vertically, and the points in the field of view (FOV) with different heights successively meet the interference conditions. For example, point A meets the interference conditions at $Z_{a}$, while point B meets them at $Z_{b}$, so $Z_{a}$ and $Z_{b}$, corresponding to the maximum value of the light-intensity envelope curve, represent the relative height of A and B, respectively, to the reference position. The relative heights of all points in the FOV are extracted to achieve the surface microscopic 3D topography by a white light interferometric data processing algorithm that combines local polarization with interpolation [18,19]. 

**Figure 3:**
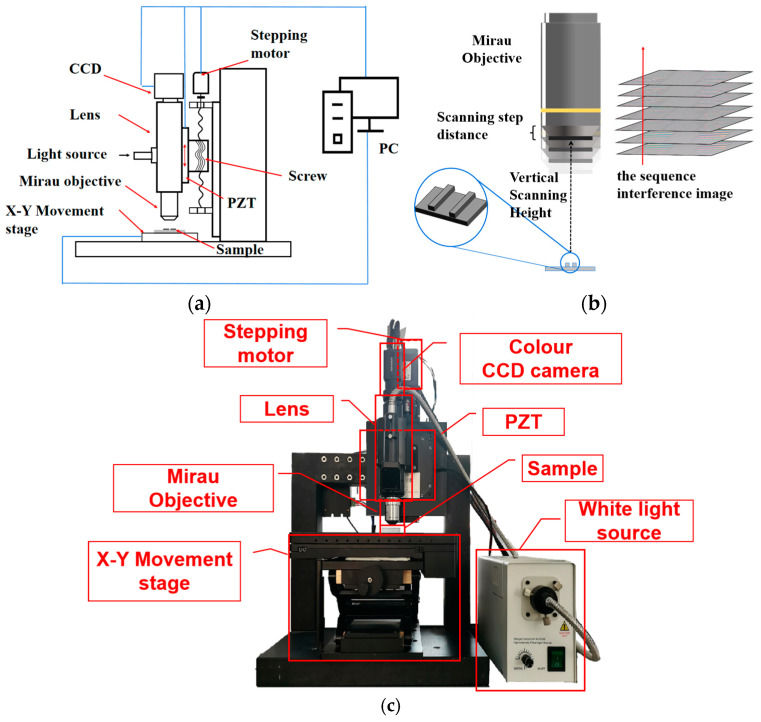
WLI measurement system: (**a**) schematic diagram; (**b**) sequence interference image acquisition process; and (**c**) the measurement system.

We adopted the MV-CS200-10GC high-resolution color surface array CCD, which has a resolution of 20 million px. The light source is a white halogen lamp with a central wavelength of 640 nm. The interference objective lens is a Nikon 20× with a numerical aperture (NA) of 0.45. The micro-displacement device PZT has a large range of 200 μm and a stepping accuracy of 7.5 nm, which can carry the acquisition module for vertical scanning. The schematic diagram shown in Figure 3a,b demonstrates the scanning process of the sequence interference images. The measurement system is shown in Figure 3c.

**Figure 4:**
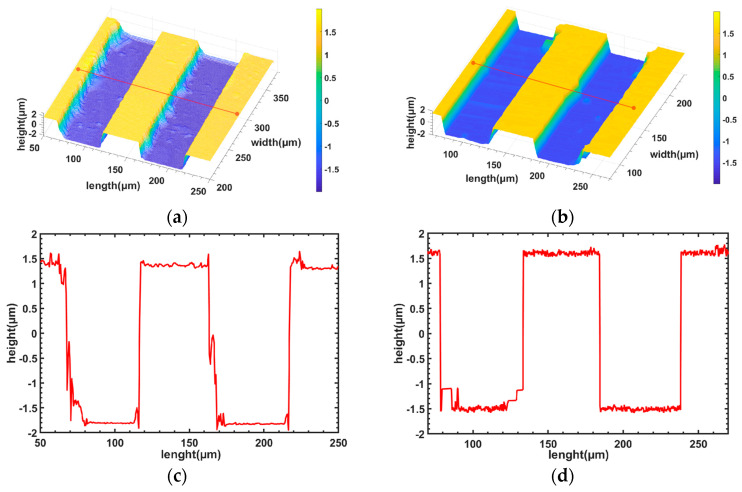
Comparison between our measurement system and the Zeiss LSM900: (**a**) step height measurement by the Zeiss LSM900; (**b**) step height measurement by our system; (**c**) cross-section of the center of the surface micromorphology of (**a**); and (**d**) cross-section of the center of the surface micromorphology of (**b**).

A laser confocal microscope LSM900 (a well-known commercial instrument for surface microform measurement) and the measurement system presented in this paper were used to measure the standard $R_{a}=1.60\;\mu m$ sample ($R_{a}$ is the arithmetic mean deviation of the assessed profile). Table 1 shows the parameter list of the confocal microscope LSM900 and our WLI system. As shown in Figure 4a–d, the average height of the steps measured by the LSM900 was 3.17 μm, and the measurement system in this paper obtained a measurement result of 3.21 μm with an error of 1.26%. As seen in the figure, the cross-sectional curves are also in better agreement, indicating that the measurement accuracy of the system in this paper meets the requirements of the surface micro-topography measurement of key components.

## 3. The Surface Characteristics That Cause Stress Concentration in Key Components and the Measurement and Evaluation Problems Arising from These Characteristics 

The surface micro-topography of the key component is composed of the surface morphology after material removal and the residue morphology left on the surface. Both of these factors affect surface stress concentration.

Equation (1) is the classical formula for the surface stress concentration factor [20].

(1) $$K_{t}=1+n\sqrt{\lambda \frac{R_{z}}{\rho }}$$

where n=1 in the torsion and n=2 in the tensile; $R_{z}$ is the height of the largest profile; ρ is the curvature radius of the valley of the notch; and λ is the ratio of notch spacing to depth. It can be seen that $R_{z}$, ρ, and λ are all decisive factors of the stress concentration. Among them, ρ and λ are related to grinding grooves after material removal, while $R_{z}$ is related to the statistical value of surface roughness. Metal chips and grinding wheel fragments may remain on the machined surface during the machining process under situations such as high-speed motion and cutting heat, causing surface defects such as smearing and adhering chips [21]. The residue morphology data attached to the machined surface are considered part of the surface and are involved in calculating and evaluating the $R_{z}$ value. However, the mechanical properties of these residues are essentially different from the substrate surface, and the residues should be treated differently when the surface is evaluated. Otherwise, they will cause errors in the stress concentration evaluation.

### 3.1. Grinding Surface Groove Is a Smooth, Highly Curved, or Tilted Surface 

**Figure 5:**
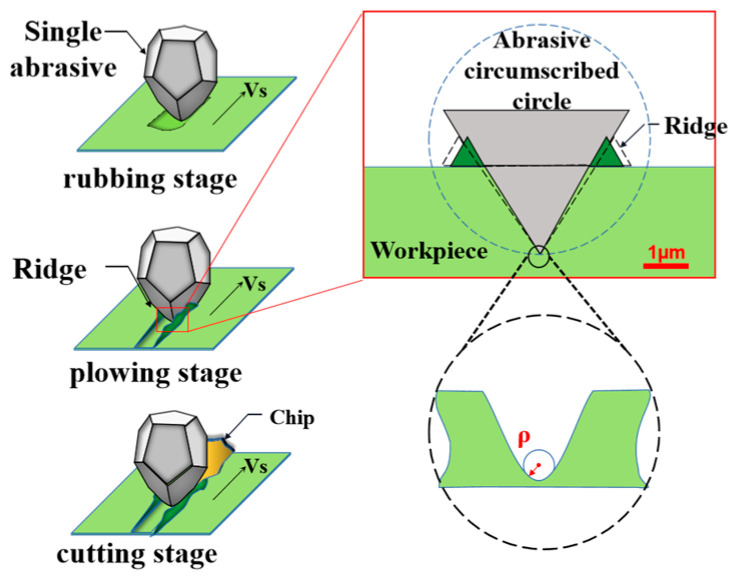
Schematic diagram of grinding grooves.

During the grinding process, the machined surface exhibits grooves that overlap with the abrasive grain contours, and the ridges generated by the lateral flow of abrasive along the plowing surface workpiece are composed of two distinct parts: (1) grooves, which coincide with the contour of the grains, and (2) ridges, resulting from the lateral flow of material along the rake face extruded by the abrasive grain [22]. In the plowing stage, the abrasive grain real-time cutting extrudes the workpiece material to form a bulge on both sides, based on which the elastic recovery deformation is caused by the grinding heat. After cooling, the cross section formed by the abrasive cutting shrinks inward. Hence, the cross-section groove angle becomes smaller, and the groove edge is steeper, as shown in Figure 5. Moreover, the radius of the groove valley is ρ, shown in Equation (1), which is the key parameter affecting the stress concentration. For a measurement task, this is a smooth, highly curved, or tilted surface (SHCTS), which is a typically difficult measurement [16]. Research [23,24] has also pointed out the difficulty of measuring SHCTS in optical micro-measurement.

**Figure 6:**
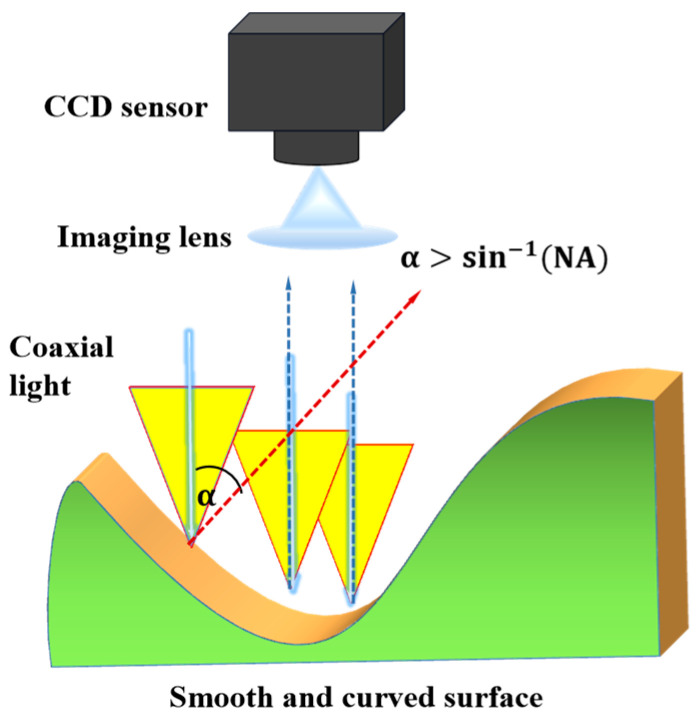
Schematic diagram of coaxial light reflection.

In microscopic optical measurement, the numerical aperture (NA) is an important parameter affecting the lens’s resolution and the through-light aperture. Since optical micro-measurement uses coaxial light illumination generally, as shown in Figure 6, the light from the coaxial light source is reflected by the surface and enters the interference lens for imaging. However, for the measurement of SHCTS, the reflected light cannot enter the optical lens when the angle between the incident and reflected beams α > sin^−1^(NA) [16], as we chose a Nikon 20× interference objective lens (NA = 0.45). Thus, when the angle α of the steep surface of the groove edge exceeds 26.75°, the reflected light will not generate an interference fringe during the scanning process, resulting in signal loss.

### 3.2. Grinding Surface Residues and Other Defects

**Figure 7:**
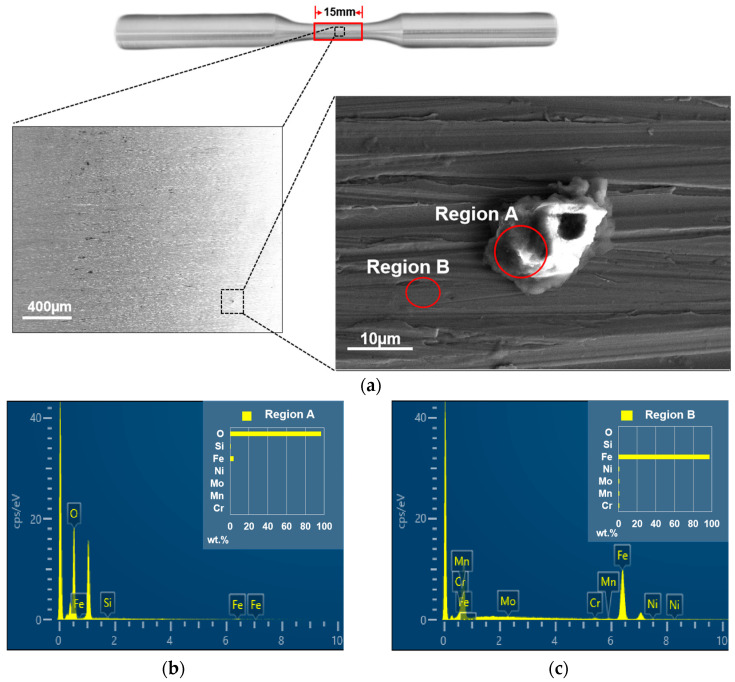
Scanning electron microscopy showing the different compositions of substrate and residue: (**a**) scanning electron microscope detection of surface residues; (**b**) Region region A; and (**c**) Region region B.

Grinding surface residues are essentially the result of cold welding during the grinding process. As the grinding process proceeds, the workpiece metal is transferred to the abrasive grain through bonding and then transferred back to the surface through the friction welding process, resulting in the formation of bonded chips on the ground and surface residues on the workpiece [21]. In fact, grinding wheel abrasive grains or bonding agents may also fall on the machined surface and bond to the surface under high temperatures and grinding forces. These residues are measured indiscriminately and are involved in the surface evaluation as part of the surface 3D topography. However, since the residues are generated under conditions of high temperature and intense metal deformation, their mechanical properties are different from those of the substrate surface. Thus, residues are an influential factor in surface stress concentrations, which should be distinguished when conducting surface stress concentration studies. In this study, the material of the measured standard test bar was 20CrNiMo steel; its chemical composition is shown in Table 2. The machining process for the test bars involved turning and grinding. A sample measuring 15 mm in length was examined by a Zeiss Crossbeam 550 scanning electron microscope, and the composition of the substrate and residue is shown in Figure 7.

## 4. Compensate Methods for Microscopic Micro-Topography Measurement and Evaluation of Key Components

### 4.1. Point Interpolation in Grinding Groove Measurement

**Figure 8:**
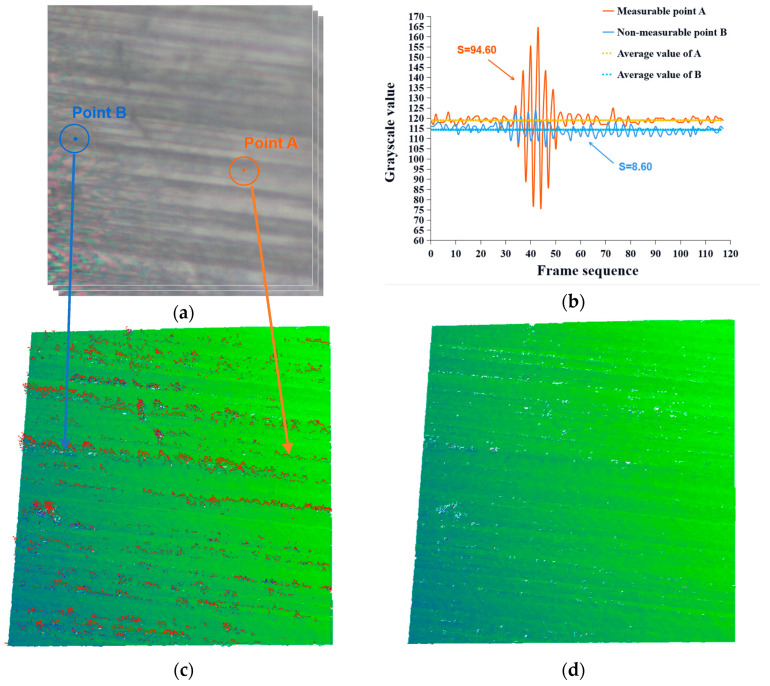
Grayscale sequence of the points on the flat and steep places: (**a**) measurable and nonmeasurable points on the grinding surface; (**b**) grayscale variation of measurable and nonmeasurable points; (**c**) point cloud data without removing nonmeasurable points; and (**d**) point cloud data after removing nonmeasurable points.

As per the analysis in Section 3.1, groove edge points cannot produce interference for imaging normally. We analyzed the gray-value sequence of point A at the flat area and of point B at the steep area, which could not be measured, as extracted from the original interferometric image sequence, as shown in Figure 8a. Compared with point A, point B had a smaller fluctuation, did not conform to the WLI signal law, and could not form an envelope curve, as shown in Figure 8b. The grayscale value sequence observed at point B was a noisy signal from a steep surface. Extracting the height value based on this noisy signal may result in an error, as shown in Figure 8c. We denoted the variance of the pixel gray value sequence as S. Based on observation and analysis of a large number of pixels, when S was less than 10, the pixel gray fluctuation did not conform to the WLI signal law. Consequently, the pixel point was classified as a nonmeasurable point and eliminated from the reconstructed point cloud, and the excluded point cloud data are shown in Figure 8d.

**Figure 9:**
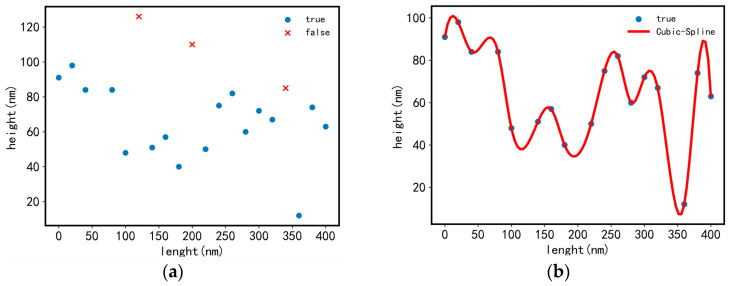
Schematic diagram of cubic spline interpolation: (**a**) before interpolation; and (**b**) after interpolation.

To provide calculated data such as the radius of curvature (*ρ*) for the stress concentration factor (*K_t_*) in Equation (1), it is necessary to make predictions (also known as interpolation or compensation) based on neighboring measurable points for the eliminated points [25]. The commonly used interpolation algorithms have been studied [25,26,27,28]. Compared with other interpolation algorithms, the interpolation error of cubic spline interpolation has been found to be smaller [25]. Therefore, the cubic spline interpolation algorithm was adopted in this paper. Cubic spline interpolation is a method of segmental interpolation with a smooth cubic function curve between every two adjacent points [29]. As shown in Figure 9a, the nonmeasurable points with distortion height were eliminated, and cubic spline interpolation was performed based on the height values of neighboring measurable points. The effect is shown in Figure 9b.

**Figure 10:**
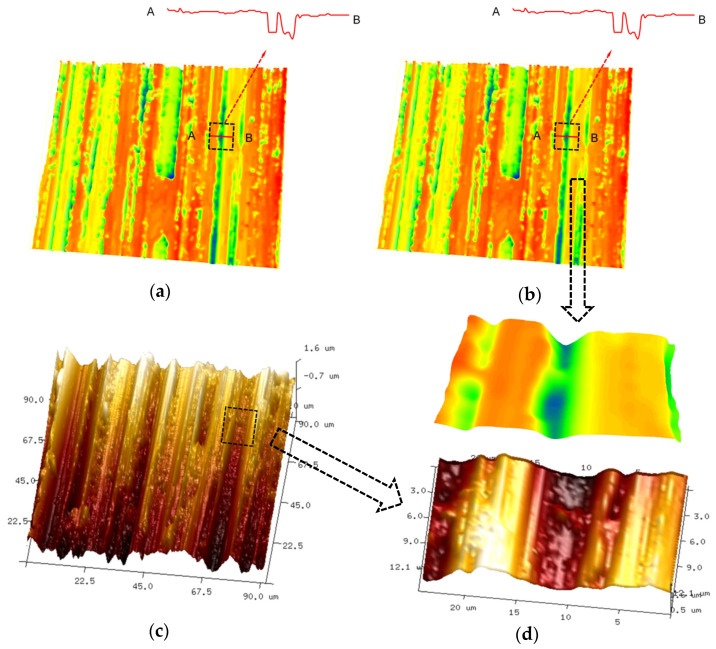
Elimination and interpolation: (**a**) point cloud after eliminating the error point; (**b**) interpolation result; (**c**) AFM measurement results; and (**d**) comparison results.

The point cloud was processed based on the abovementioned elimination rule and interpolation algorithm. As shown in Figure 10a, the distortion points were eliminated. Eliminated points were interpolated, as shown in Figure 10b. It is shown in Figure 10a that, owing to the elimination rule, distortion points were eliminated between points A and B. The predicted compensation result of AB is shown in Figure 10b. To verify the validity of the elimination rule and interpolation algorithm, we used the Bruker Dimension Icon Atomic Force Microscope (AFM), a 3D micro-topography measurement instrument based on the contact measurement mode, wherein no information is lost at the steepness, to measure the same surface, as shown in Figure 10c. The measurement data matched the interpolation results well, as shown in Figure 10d. The comparative results demonstrate that the proposed method of nonmeasurable point rejection and interpolation compensation had a good reconstruction effect on the steep grinding groove morphology that caused stress concentration on the surface of key components. In addition, both the point cloud with nonmeasurable points eliminated and the interpolated point cloud can be provided to researchers of surface stress concentration on critical components for surface evaluation and calculation of the surface stress concentration factor.

### 4.2. Intelligent Identification and Segmentation of Surface Residues and Other Defects

As described in Section 3.2, residues generated by grinding that have different mechanical properties from the substrate should be distinguished and segmented for residue information extraction. This information provides a database for surface evaluation of key components when studying surface stress concentrations. However, conventional WLI measurements only provide the point height value, while the residues are distinguished from the surrounding substrate in coloration. Therefore, to extract and segment the residues, we established a practical approach to obtaining the point cloud and super-depth-of-field image simultaneously for residue information extraction.

**Figure 11:**
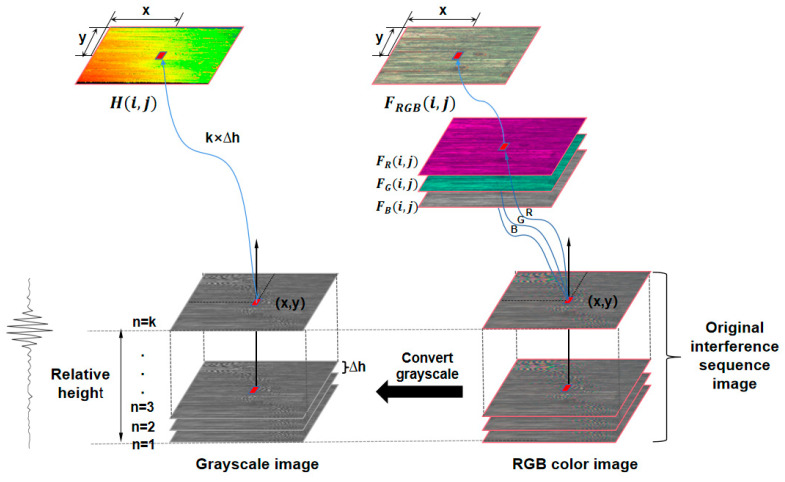
Principle diagram of simultaneous acquisition of point cloud and super-depth-of-field fusion images.

As in Figure 11, a colored CCD was used to capture a sequence interference image and replicate two versions, where the left sequence was grayscale images converted from RGB ones, and the point cloud H(i,j) was extracted based on grayscale images following the principles in Section 2. A super-depth-of-field fusion algorithm was applied to the right sequence images, as shown in Equation (2), to obtain a super-depth-of-field image.

(2) $$F_{RGB}\left(i,j\right)=max\left\{A_{1}\left(i,j\right),A_{2}\left(i,j\right),\ldots ,A_{n}\left(i,j\right)\right\},$$

where $F_{RGB}\left(i,j\right)$ is the RGB fusion array; $A_{1},A_{2},\ldots ,A_{n}$ is the original sequence image; and i and j are the row and column numbers of pixels in the image, respectively. $F_{R}\left(i,j\right)$ is the maximum of the pixel (i,j) R-value sequence; $F_{G}\left(i,j\right)$ is the maximum of the pixel (i,j) G-value sequence; and $F_{B}\left(i,j\right)$ is the maximum of the pixel (i,j) B-value sequence. Merging the three channels $F_{R}\left(i,j\right)$, $F_{G}\left(i,j\right)$, and $F_{B}\left(i,j\right)$, we could obtain the super-depth-of-field image $F_{RGB}\left(i,j\right)$. Clearly, $F_{RGB}\left(i,j\right)$ and H(i,j) were obtained simultaneously, the point cloud and the super-depth-of-field image had the same coordinate reference, and the information of the two entities could complement each other.

**Figure 12:**
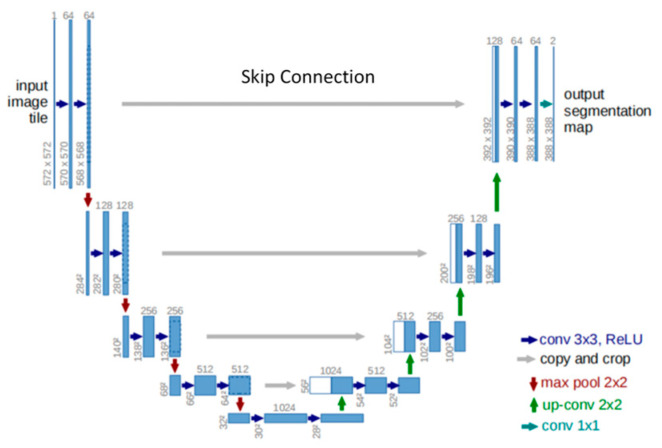
Structure of a U-net neural network.

Based on the super-depth-of-field image with pixel color information, further residue segmentation can be performed to obtain residue information. However, owing to the complex texture of the grinding surface and the various patterns of residues, it is difficult to implement traditional image segmentation algorithms. Considering that the U-Net semantic segmentation network has been successfully applied to various scenarios (e.g., industrial defect detection, satellite image segmentation) with an outstanding segmentation effect, we used U-Net for residue semantic segmentation in this paper [30,31]. The U-Net network architecture is a symmetric encode-decode structure, as shown in Figure 12, with an encoder on the left and a decoder on the right. The encoder uses convolution and down-sampling to obtain the feature mapping, extend the acceptance field, and integrate the context information. The decoder uses up-sampling and deconvolution to decode the feature map and then outputs the pixel-level classification results with the same size as the original image to achieve semantic segmentation. U-Net innovatively adopts a skip connection to supplement the information by concatenating the feature map in the up-sampling process and the feature map with the same size in the down-sampling, taking into account both low-resolution information (for target class recognition) and high-resolution information (for precise segmentation and localization). Owing to data augmentation with elastic deformations, U-Net can make more efficient use of limited annotated samples [32]. As a result, U-Net can achieve good segmentation performance even with a small number of annotated samples. 

**Figure 13:**
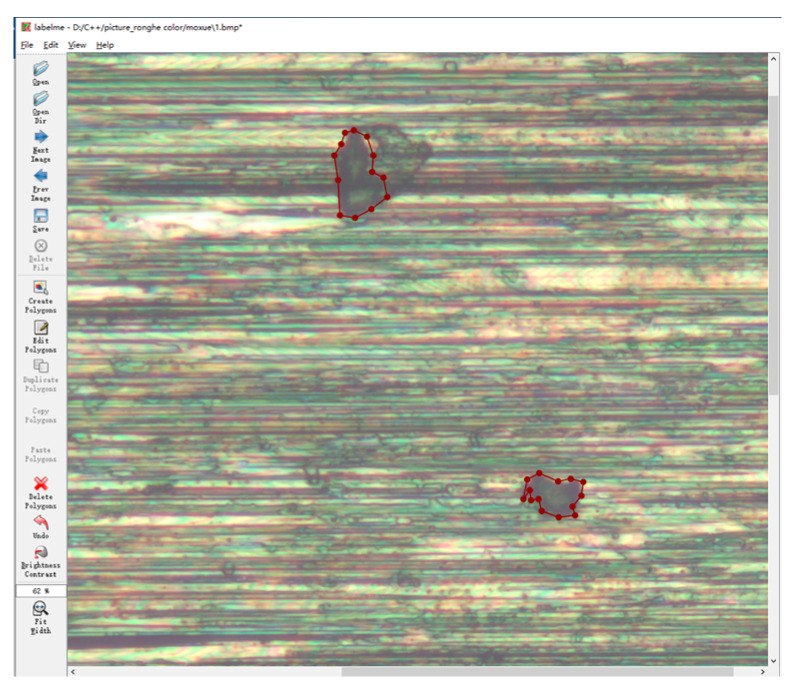
Label making.

**Figure 14:**
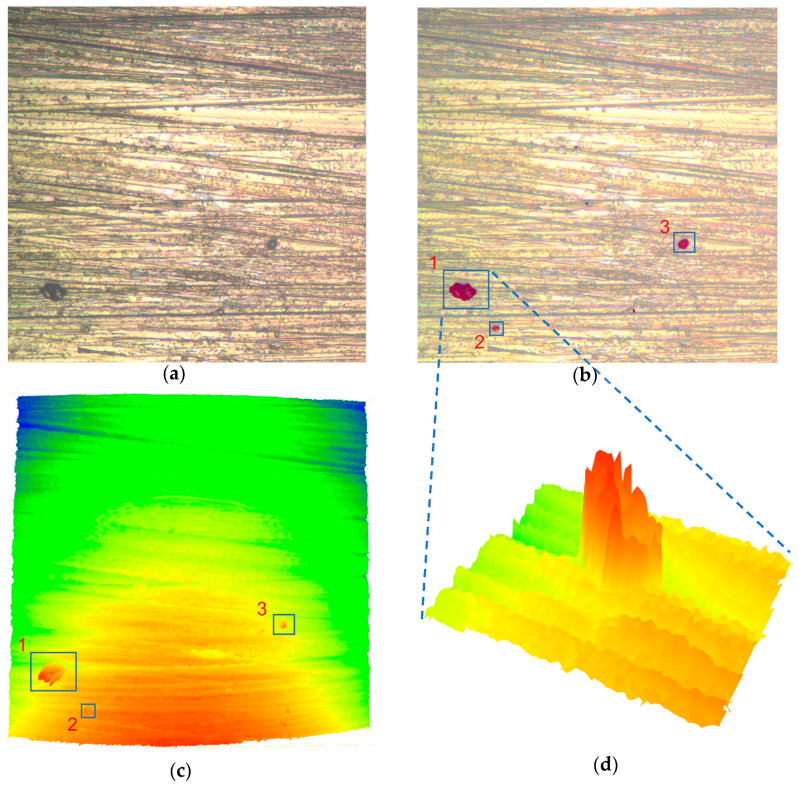
Semantic segmentation of surface residues and point cloud extraction: (**a**) original super-depth-of-view image; (**b**) semantic segmentation of residues; (**c**) corresponding point cloud; and (**d**) semantic segmentation of residues.

Figure 13 shows the label creation using LabelMe for residues and other defects. Defect samples were collected from a set of 100 super-depth-of-field images, which were used for training after data enhancement. The ratio of the training, validation, and test sets was 60%:20%:20%. The training hyper-parameters are shown in Table 3. The final prediction accuracy of 91.06% was obtained. Figure 14a,b shows the original image and prediction results. Since the super-depth-of-field image and the point cloud were taken from the same field, both had the same XY coordinate system, so it was easy to locate the residues in the point cloud based on the connected domain segmented by U-net in the super-depth-of-field image, as shown in Figure 14c,d. The distribution location, area, and equivalent diameter of each residue are shown in Table 3. The relative height difference of the residues relative to the substrate is also listed in Table 4. This information can provide a valuable reference for the scientific evaluation of key component surfaces.

## 5. Conclusions

Grinding grooves and residues on the key component surface cause stress concentration. In this paper, we researched the measurement and evaluation methods for grinding grooves and residues.

1. For the problem of the signal distortion on the side of the grinding groove, we proposed setting a pixel gray-value threshold to exclude the distortion points and adopting the cubic spline interpolation algorithm to compensate. The compensated grinding groove was compared with the measurement data of AFM, and the result shows a high match.
2. For the surface residues that are difficult to distinguish by ordinary instruments, the super-depth-of-field image and the corresponding point cloud were obtained from the same WLI image sequence synchronously. The semantic segmentation network U-net was used to segment the residues on the super-depth-of-field image and obtain an accuracy rate of 91.06%. Based on residue segmentation in the super-depth-of-field image, residue information could be extracted, such as their height and location in the point cloud.

The solution proposed in the paper has constructive significance for measuring and evaluating the micromorphology of the surface of key components. It can provide a data basis for the study of surface stress concentration. Owing to the large calculation for interpolation of distortion points, we will adopt phase-shifting interferometry (PSI) or the BEM optical scattering model in future work to study whether it can reduce the distortion points of the grinding groove to reduce the calculation. Moreover, for surface defects, we will further study the identification of other defects, such as burns, to provide a more comprehensive data basis for surface stress concentration evaluation.

## Figures and Tables

**Table 1:** Parameter list of confocal microscopes (LSM900) and our WLI system.

Parameter	LSM900	Our WLI System
Magnification	20	20
Horizontal Resolution	0.12 μm	0.69 μm
Vertical Resolution	10 nm	7.5 nm
Field of View	400 μm × 400 μm	245 μm × 306 μm

**Table 2:** Chemical composition of 20CrNiMo steel.

Element	C	Si	Mn	S	P	Cr	Ni	Mo	V	Nb	Fe
wt.%	0.21	0.25	0.78	0.004	0.009	0.61	0.58	0.24	-	-	rest

**Table 3:** Training hyper-parameter settings.

Hyper-Parameters	Image Width	Image Height	Batch Size	Image Scale	Learning Rate	Optimizer
Value	512	512	2	0.7	1 × 10^−3^	RMSProp

**Table 4:** Residue information.

Grain Number	Area (μm^2^)	X (μm)	Y (μm)	Equivalent Diameter (μm)	Relative Height Difference (μm)
1	557.9	46.50	302.18	29.77	2.34
2	49.41	82.48	342.98	8.49	1.67
3	153.98	280.95	252.77	14.95	1.98

## Data Availability

Not applicable.
